# The Hospital. Nursing Section

**Published:** 1902-07-05

**Authors:** 


### The Hospital.

### Nursing Section.

Contributions for this Section of "THE HOSPITAL" should be addressed to the EDITOR, "THE HOSPITAL" NURSING SECTION, 28 & 29 Southampton Street, Strand, London, W.C.

No. 823.—VOL. XXXII. *SATURDAY, JULY* 5, 1902.

### Notes on News from the Nursing World.

### THE QUEEN AS NURSE.

NEXT to intense anxiety concerning the King, the predominant feeling of the nation has been one of profound sympathy with the Queen, who, from the hour that the illness of his Majesty was announced to the moment when he was declared to be "out of immediate danger," has had imposed upon her the heaviest of burdens. In a very real sense she has shared the duties of nursing her Consort. While a nurse from the London Hospital—Nurse Haines—who had already acted as nurse in the Royal Household, and a staff nurse from Miss McCaul's private nursing home in Welbeck Street—Nurse Fletcher—have been in attendance upon the Sovereign since the operation, at which they were present, the Queen has been frequently in the sick-room, ministering to the needs of her husband, yet not in any way interfering with the trained nurses in the discharge of their responsibilities. If her presence has been like sunshine to the Royal sufferer, her unceasing solicitude and sympathy have had an encouraging influence upon those who were charged with the task of tending him through the critical period when the nation waited, with breathless suspense, for the issue of bulletins from Buckingham Palace.

### THE ROYAL RED CROSS.

To nurses the most interesting feature of the long list of Coronation Honours which was published on Thursday last, is the South African portion containing the names of the ladies upon whom the King has been graciously pleased to bestow the decoration of the Royal Red Cross. At the head of these is Miss E. M. Chadwick, superintendent of the Army Nursing Service, with the Hospital Ship *Maine*, and then follow the names of Miss M. C. S. Knox, of the Army Nursing Service; Miss Elizabeth Cochrane Shannon, and Miss H. Hogarth, of the Army Nursing Service Reserve; Miss Frances Amelia Lowrie, matron of Bourke Hospital, Pretoria; Miss Ada Whiteman, matron of the Leper Hospital, Pretoria; Miss Lucy Alice Yeatman, and Mrs. Redpath, Colonial Nurses, Ladysmith; Mrs. Melina Rorke, Colonial Nurse, Bulawayo; and Miss Ethel McCaul. The decoration has also been awarded to Mrs. George Cornwallis-West, of the Hospital Ship *Maine*; Mrs. Theodosia Bagot, of the Portland Hospital; Mrs. T. C. Wolley Dod, Pretoria, Mrs. Keith, Pretoria, and Miss Knight, Care of Prisoners of War, Pretoria; Mrs. P. H. Johnston, President, Army Hospital Aid Society, Natal; Mrs. Jessie Burrill and Mrs. Emma Francis, Durban Women's Patriotic League; Mrs. Jane Waterston, Mrs. Sclater, Mrs. Kilpin and Mrs. Ball, Good Hope Society, Natal; and Georgina, the Dowager Countess of Dudley. It is also announced that Miss Martha Thomas, Miss Sarah Emily Webb, Miss Sarah Elizabeth Oram, and Miss Mary Cecil Florence Kate Cole, Superintendents of the Army Nursing Service, and Miss Louisa Watson Tulloh, Sister of the Army Nursing Service, have been enrolled as Honorary Associates of the Grand Priory of the Order of the Hospital of St. John of Jerusalem.

### NURSES AND THE CORONATION.

THE general disappointment felt at the postponement of the Coronation was shared by the nurses of the hospitals on the line of route. Stands had been erected in most cases, and special seats were allotted to the nurses. At Westminster, great preparations had been made, and many of the wards were closed. Those nurses who were away for their holidays were intending to spend a night at the hospital, and to witness the procession, returning home again next day. The cost of the stands was very large—probably two or three thousand pounds—and a circular is being issued to seatholders on the subject. Some who had taken seats have generously given the whole amount towards the funds of the hospital, and to others 10 per cent. will be charged on the amount repaid. This, however, does not cover the cost. The refreshments were being catered for, and were insured. Charing Cross and St. George's Hospitals have also issued notices respecting the seats. At St. Thomas's, the front of which was finely decorated, a large red and white notice reminded the public that this was the first training school for nurses, founded in 1857, by public subscription as a testimonial to Miss Nightingale.

### QUEEN ALEXANDRA'S IMPERIAL MILITARY NURSING SERVICE.

THE following Army Order relating to the formation of the Queen Alexandra's Imperial Military Nursing Service has been sent to us from the War Office:—(1) All present members of the Army Nursing Service, and members of the Army Nursing Reserve who have been in military employment during the war in South Africa, shall be eligible for appointment in the Queen Alexandra's Imperial Military Nursing Service, if recommended by the Nursing Board. Should any question arise as to their status in the Queen Alexandra's Imperial Military Nursing Service, the Nursing Board shall report thereon to the Advisory Board, and the recommendation of the Advisory Board shall be submitted to the Commander-in-Chief, whose decision shall be final. (2) Any present member of the late Army Nursing Service, who is not retained in the Queen Alexandra's Imperial Military Nursing Service, may be recommended for a gratuity of one month's pay for each year of service if she is not entitled to a pension; and any nurse accepted by the Nursing Board as a member of the Queen Alexandra's Imperial Military Nursing Service who may decline to accept the new terms of pay, pension, etc., shall be allowed to serve upon the terms of her present engagement.

### THE COLONIAL NURSING ASSOCIATION.

Princess Henry of Battenberg will be present at the annual meeting of the Colonial Nursing Association to be held on Wednesday afternoon next, by kind permission of Her Royal Highness, at Kensington Palace, and Earl Grey will preside. The sixth annual report of the Association shows that 32 Government and 9 private nurses were sent out, an increase of 11 during 1901; that the total number of nurses now at work is 89 as against 67 last year; that two new branches have been formed in Oporto and in the Falkland Islands; that of the new nurses sent out for Government work five were appointed by the Malay Federated States to work either in hospital or as private nurses; that a nurse matron has been provided for St. Vincent, and that a nurse has been selected for St. Lucia. Both the medical superintendent at Sierra Leone and the chief medical officer at Accra testify to the benefit derived by the residents from the introduction of skilled European nurses. The death of one nurse, Miss Isabella John, at the Government Hospital, Gold Coast, is the only sad item of intelligence. As to the financial position, the invested fund has now almost reached the sum of £5,000 suggested by Mr. Chamberlain, but it is pointed out that if the work of the Association is to continue to develop in accordance with the demands made upon it and the increased appreciation shown by the Colonies, it will be necessary for the annual subscription list "to be very much enlarged." The Committee acknowledge with gratitude a donation of £500 from the Government of the Malay Federated States, which the Acting High Commissioner describes as "a mark of appreciation of an institution formed to ameliorate the condition of life in distant parts of the Empire."

### DEATH OF SISTER COTTON OF GUY'S.

We regret to have to record the death of Sister Clemence Margaret Cotton, eldest daughter of the late Lord Justice Cotton, who died at Guy's Hospital on June 23rd. The funeral service was held at Guy's Hospital Chapel on Saturday, when the building was filled with mourners and the staff of the hospital. There was a large number of most exquisite wreaths and crosses. The interment took place at the family burial place at Liphook in Hampshire. Miss Cotton began her training at Guy's Hospital in 1891, and there all her nursing work was done. It was found necessary about a fortnight ago to perform an internal operation, which was entirely successful, but constitutional weakness asserted itself and Miss Cotton passed away about 10 days later. Those who knew the devotion which she threw into her work will readily understand how great is the loss the hospital has sustained. As head of the Lying-in and Gynæcological Departments she had especially endeared herself to the women and girls who passed through the hospital.

### CONSIDERATION FOR THE NURSES.

In the new children's wards at the Beckett Hospital, Barnsley, which was opened by the Mayoress on June 27th in the presence of a very large gathering, not only has the comfort of the seventeen little inmates been considered in every way, but the nurses have also been studied. All the latest sanitary appliances are to be seen in the lavatories, but a special and noteworthy feature is the small bath raised to a considerable height so as to render stooping on the part of the nurse unnecessary. The furniture of the ward is thoroughly modern in type. The cots are enamelled white, and have brass fittings; the furnishings are made of selected pitch-pine, and that the tiny colony may not be without musical consolation a handsome piano has been presented to the ward. The British Women's Temperance Association has promised to provide a cuckoo clock. The building is entirely the outcome of the efforts of the Hospital Saturday and Sunday Committee, and a considerable portion of the hundreds of pounds which have been spent on this undertaking was gathered together by penny collections among school children—thus the ward is a children's ward in more senses than one.

### ENGLISH NURSING IN ROME.

The letter which we publish from "M.F.D." this week shows how superficially she reads The Hospital. In criticising an article by a contributor on "English Nursing in Rome," which appeared in our issue of June 14th, she accuses us of being "obviously unjust to Catholics." If she perused our columns regularly she would have known that on more than one occasion lately we have been obliged to condemn Protestant bigotry, and have even been called over the coals for Roman Catholic predilections. The introduction of the *odium theologicum* into nursing is as objectionable as it is unnecessary, and we have always strongly deprecated it. With regard to the article to which "M.F.D." refers, we do not think that the author merits the censure which she has provoked, or that she intended to attack the English nuns. It would have been better if, in her introduction, she had explained that she used the words "nursing community" in the plural sense, viz. that the nursing communities were all under religious orders or belonged to such. She would also have been on safer ground if, instead of attributing want of trained knowledge to the nurses, she had merely suggested that the training of the nurses in the Anglo-American Nursing Home is more in accordance with English and American ways. We think, too, that she rather invited retort by her statement that the Roman Catholics or religious orders, in Rome "wanted to keep the nursing element in their own hands." But, for the rest, we can only say that, in our judgment, the article seems a perfectly temperate account of a movement which is necessarily interesting to nurses, especially to those who contemplated joining the staff of the Anglo-American Nursing Home.

### THE RAILWAY COMPANY AND THE NURSES' HOME.

We warmly congratulate both the treasurer of the Royal South Hants and Southampton Hospital, and the directors of the London and South-Western Railway Company on the exceedingly generous assistance given by the latter to the fund for a Nurses' Home at Southampton. When the directors of this company were first approached in the matter, they stated that the circumstances would not permit them to subscribe to the fund; but it being pointed out that the relationship between Southampton and the company was entirely different to that of any other town on the system, Mr. Owens, the general manager, replied that he had much pleasure in intimating that his board had "unanimously voted the sum of £500." It would, of course, be quite unreasonable, in ordinary circumstances, to expect a railway company to help in such a case, but Southampton contains so many employés of the South-Western Company to whom the doors of the Royal South Hants Hospital are always freely open, that we can understand why the appeal was made, and why, on reconsideration, it was responded to. But the liberality of the response was a welcome surprise, and may be commended as an example to other railway companies who are under any similar obligation.

### NIGHT NURSES ON THE HOSPITAL SHIPS.

The arrangements for the conveyance from the shore of the supper of the nurses employed on the Hospital Ships at Dartford are far from satisfactory. According to information which we have received, a nurse has to carry meat on a dish, weighing together from 17 lbs. to 20 lbs., a distance of some 300 yards before reaching her destination, in all sorts of weather. Immediately afterwards, she is expected to carry the patients' suppers round the wards. Another complaint is that if a nurse is excused from breakfast on account of headache or exhaustion, she is not permitted to have a cup of tea in her bedroom. One of the regulations is that nurses are not allowed to walk on deck, and they think that when they have been on duty in the stuffy wards all night they might be permitted to enjoy this privilege for a few minutes in the morning. As the work on the Hospital Ships at night is wearing, the authorities would consult their own interests, as well as those of the nurses, by making concessions that are clearly practicable.

### A FORWARD MOVEMENT IN PARIS.

A committee has been nominated by the Assistance Publique to examine a scheme recently laid before the Paris Municipal Council which includes certain proposals concerning the nursing staff. M. Mourier, the director of the Assistance Publique, favours the substitution of female nurses for male nurses, and he proposes to supply them in the future by the establishment of one training school in place of those at present existing at the Salpêtrière, Bicêtre, the Pitié, and the Lariboisière hospitals. It is a part of the scheme that the nurses should be trained for two years before being permitted to serve in one of the hospitals. This seems to be a move in the right direction, and under the new order of things it may be assumed that the long hours of nurses at the municipal hospitals will be reduced and the atrocious sleeping accommodation improved.

### WHOLESALE POISONING BY AN AMERICAN NURSE.

Happily, it is rarely indeed that anyone runs the risk of being poisoned by a qualified nurse, either through misadventure or intentionally. But a ghastly story is told of the terrible crimes of Jane Toppan, an American trained nurse. This wretched woman, who was tried last week at Barnstable, Massachussetts, confessed, with a smile, that she had poisoned 31 persons. Her explanation was that she "could not control her impulses." Toppan, who is 44 years of age, and is described as "not of attractive appearance," then proceeded to name her victims, and narrated how she administered morphine to them, "so that their lives were slowly slept away." "When the paroxysm passed," she said, "I was myself again. I cared no longer for the patients to die. Then my great thought was to resuscitate them. I sent for doctors and other nurses and tried my best to save them. Sometimes I was successful, but on many occasions the poison was too much. I used morphine and atropine because vegetable poisons can hardly be detected, even before death." Still, it is an extraordinary thing that she was not found out. The judge held that she must be insane, but she affirmed that she "never failed to realise what she was doing." This admission, however, did not prevent the jury from endorsing the theory of the judge, and the wholesale poisoner was committed to a lunatic asylum for life. There, it may be hoped, she will have no chance of giving way to her appalling impulses. Let us also hope that this horrible confession is itself, in part at least, the outcome of an insane delusion.

### VISITING NURSES FOR PAYING PATIENTS.

A month ago we mentioned a movement in Marylebone to provide a visiting nurse to attend patients in the district of Marylebone by the hour, and suggested that the promoters should either decide to employ more than one nurse, or limit the visits of the single nurse to a portion of the huge borough. It is evident from the number of letters we have received from correspondents who desire employment of the kind that the organisation would not find it difficult to enlarge their staff sufficiently to afford the movement an adequate chance of success. In order that our correspondents may have the opportunity of offering their services to the Marylebone Daily Visiting Nursing Association, we may state that the hon. secretary is Mrs. S. Spring Rice, 1 Bryanston Place, W.

### ANOTHER APPOINTMENT NEEDED AT ARMAGH.

The nurse who was chosen by the Armagh Board of Guardians for their fever hospital—this being the third time of making an appointment—has refused to comply with the request of the Irish Local Government Board to submit herself to examination for a certificate of qualification in nursing, and yet another election has to take place. We conclude that if Miss Frith's credentials as to training had been in accordance with the regulations of the Board, they would not have made the stipulation for an examination on their own account. She, however, claims to be in possession of a certificate "considered sufficient for a good appointment in England." The latter she says she refused, "giving the Armagh Board of Guardians the preference." But she would have been more prudent if she had previously ascertained the conditions she would have to fulfil in order to be confirmed in the appointment at Armagh.

### PROCRASTINATION AT COLCHESTER.

It is not easy to understand why the Colchester Board of Guardians did not accept the motion of Mr. Peck for the appointment of an assistant nurse. With 113 patients in the workhouse infirmary tended by five nurses, it is clear that the requirement of the Local Government Board, which is that there should be one nurse to every fifteen cases, is not fulfilled. We agree with Mrs. Hay as to the importance of the proposed additional nurse being certificated, but we cannot see that there was any reason to waste time by referring the matter to the house committee, when the late superintendent nurse, the doctor, and the Local Government Board are of one mind as to the necessity for further help.

### Lectures to Nurses on Medical Nursing.

By Eveline A. Cargill, M.D.Brux., L.R.C.P.&S.Ed.

### LECTURE I.—INTRODUCTORY.

This course of lectures was primarily intended for district nurses, though by no means confined to them ; still, perhaps, it is in district nursing that an occasional stimulus, such as this course is meant to be, is most valuable. Those of you who have been doing district work for some time must feel, to a certain extent, cut off from the world of medicine, apt to get a little rusty, to forget first principles, to get, perhaps, a little mechanical. You cannot see new methods of treatment, new appliances; you have not much time for reading in your busy active life, and sometimes it must be difficult to keep up your professional enthusiasm. Then in district work the doctor is always in a hurry; he cannot spare time to teach you, to explain the case; but the district nurse is thrown on her own resources, and has to think and act for herself, and worry through her own difficulties and perplexities as best she may. So that among all nurses the district nurse should have scientific principles to guide her in her work, and as you cannot all go into hospital from time to time to get rubbed up, a course of lectures must serve the purpose.

There is another aspect of district work that is peculiarly trying to the nurse, and that is the depressing nature of many of the cases she is called on to attend; there must necessarily be a large proportion of chronic and incurable cases, and these in circumstances that admit of little amelioration. There is something invigorating about a really acute case, you feel you have a good battle before you, and we all enjoy a fight. And then the triumph when the patient is pulled through! it makes up for the hard work and anxiety. But in chronic cases all this excitement (and how should we get through life without excitement?) is absent, and you feel almost as if you were doing no good, that the fight against poverty, disease, and death were too much for you, that the odds against you were too great, and that because there was not much to show for all your work that therefore it must be unproductive. It is almost impossible not to feel this discouragement at times when working among the poor, and here again a little help from outside is stimulating, and encourages you to see your work in its true proportions.

The poor we shall have always with us, but we need not always have the unnecessary adjuncts of poverty, namely, dirt and vice. Here the nurse may know for certain she is doing good work, for her attitude towards vice of all kinds is a wholesome influence, and most certainly is a great force for good. It is not necessary to "preach" in order to exert this right influence; indeed "preaching" often weakens the cause, which a quiet, steady, earnest attitude will strengthen, and the nurse has more opportunities of exerting that influence than even clergyman or doctor, for she comes into closer contact with the individual when weakened and softened by illness.

Also in the war against dirt of all kinds, by which I mean defective personal hygiene, house sanitation, disposal of refuse, infection, and the whole range of defective sanitation, the nurse is a pioneer, and can do more than anyone else to instil into the minds of the poor the evils of dirt and the advantages of cleanliness. In fact, the nurse, in addition to her nursing capabilities is a health missioner and has endless opportunities of teaching, of enlightening the ignorance and rebuking the carelessness of our poorer neighbours. Indeed, district nursing is work of the highest practical utility, bringing an elevating and purifying influence into homes otherwise unreachable and fighting successfully the war against ignorance, disease and evil.

To be a good nurse a woman must be intelligent, and the more she cultivates her intelligence the better nurse she will be; both in district work and in private nursing she must constantly fall back upon her own reason and knowledge, and therefore must be much more than a "machine," slavishly obeying orders. Perhaps even of more importance is a kindly and sympathetic nature, but kindliness and intelligence must go hand in hand. A woman with no kindliness, no sympathy with weak suffering human nature may do in other occupations, but she will never make a nurse. On the other hand, though the sympathetic nature be there it must not be allowed to come too much to the front, partly because expressed sympathy is not always welcomed by the patient, and partly because hyper-sensitiveness in any direction is apt to hinder her own work; to allow the mind to dwell too much on suffering tends rather to encourage weakness, and a nurse must be strong. She must be also cheerful and confident, quick, resourceful, firm and observant. The "seeing eye" is most essential, and the faculty of observation should be cultivated, to note every change in the patient in expression, movement, position, colour, etc., as well as the special points connected with disease to be dwelt on later. In no profession is correct observation so important as in medicine, and the nurse should learn to "observe" in order to report to the doctor as well as for her own guidance. As to firmness, a good rule is never to press a point that is not necessary, give in to the patient in all non-essentials, but when a thing is really important you must carry it through, and here tact and resourcefulness are indispensable.

Now a nurse has to do the very best possible for the patient under the doctor's orders, and the relationship between doctor and nurse should be one of mutual respect and courtesy, remembering that the doctor is in the position of superior officer, entitled to strict obedience in all professional matters. And the doctor in return should see that the nurse is so placed that she can carry out his orders, that her food, rest, exercise, etc., are provided for, and that she receives the consideration that is necessary to enable her to work well. This refers specially to private nursing when the patient's friends are often inconsiderate, and expect a nurse to be on duty day and night, forgetting that her health is her most valuable possession. On the other hand the nurse should remember that "a trained nurse" is often a great tax on the resources of people of limited means added to the other expenses of a severe illness and should not be exacting, or expect luxuries. Good plain food, a clean, quiet bedroom, and "time off" for rest and exercise, are all she must ask, while she should try to accommodate herself in little things to the ways of the household.

And though the nurse must remember that as long as she is in charge of a patient she represents medical authority in the absence of the doctor and must guard the patient's (medical) interests, still her authority should be upheld with tact and gentleness and not by expressively asserting it. Here again I would urge the nurse to yield whenever yielding does not affect the patient injuriously.

In conclusion I would press on the nurse the great importance of the care of her own health. For good health means good mental power, capacity to deal with emergencies as well as the ordinary routine work. Do not neglect small ailments or cut short your sleep or exercise. Rest whenever you can. Your work is often arduous, and makes great demands on your strength, therefore rest when there is nothing special to do that when the call for exertion comes you may be ready to meet it.

### Queen Victoria's Jubilee Institute for Nurses.

HER MAJESTY QUEEN ALEXANDRA has been graciously pleased to appoint the following as "Queen's Nurses," to date July 1st, 1902:—

ENGLAND.

| Nurse. | District Training at | Working at |
|---|---|---|
| Annie E. Penrith | Central Home, London | London |
| Margaret Murphy | Chelsea | Ditto |
| Ethel Mary Blackburn | Shoreditch and Bethnal Green | Ditto |
| Margaret Malcolm | Ditto | Ditto |
| Mary E. Morton | Ditto | Ditto |
| Hannah N. M. Williams | Ditto | Ditto |
| Gertrude Von Woerden | Hammersmith | Ditto |
| Julia Davies | Cardiff | Ditto |
| Rosa Friel | East London | Ditto |
| Kate Emily Fox | Central Home, London | Amersham |
| Lesley E. Lewis | Ditto | Bideford |
| Margaret Myles | Liverpool | Birkenhead |
| Mary H. Jones | Birmingham | Birmingham |
| Kate B. Harrison | Ditto | Ditto |
| Bertha Sawyer | Bolton | Bolton |
| Frances L. Ackermann | Cardiff | Boxgrove |
| Ethel Nichols | Brighton | Bridgwater |
| Mary E. Bennett | Ditto | Brighton |
| Edith M. Mabey | Cardiff | Carisbrooke |
| Emily K. Rawlings | Coventry | Coventry |
| Caroline Sowden | Liverpool | Crook |
| Marianne York | Brighton | Darwen |
| Norah M. Terry | Shoreditch and Bethnal Green | Deerness Valley |
| Annie Tyson | Ditto | Ditto |
| Emily Patello Pirie | Westminster | Doncaster |
| Annie Ringrow | Portsmouth | Fletton |
| Emily W. Watson | Gateshead | Gateshead |
| Mary May Merrett | Portsmouth | Gloucester |
| Annie Rivett | Ditto | Ditto |
| Ella Sissons | Walworth | Hanley |
| Lucy A. Hoult | Bermondsey | Haslingden |
| Harriett P. Moore | Ditto | Hebden Bridge |
| Elizabeth A. Ward | Central Home, London | Hertford |
| Louisa C. C. Wood | Southampton | Huddersfield |
| Frances Morgan | Central Home, London | Kettering |
| Mary A. S. Wells | Ditto | Ditto |
| Julia E. Lloyd | Gloucester | Leamington |
| Mary H. Bishop | Central Home, Liverpool | Liverpool |
| Edith Goodwin | Ditto | Ditto |
| Elizabeth Peacock | Ditto | Ditto |
| Ellen Skelton | Ditto | Ditto |
| Laura A. Noble | Kirkdale Road, Liverpool | Ditto |
| Sara Glass | Ditto | Ditto |
| Louisa Graham | Overton Street, Liverpool | Ditto |
| Margaret E. Ayre | Central Home, London | Loughborough |
| Emily L. Taylor | Bradford, Manchester | Manchester |
| Mary Kane | Harpurhey, Manchester | Ditto |
| Clara M. Hogarth | Hulme, Manchester | Ditto |
| Florence M. Russell | Ditto | Ditto |
| Kathleen Ryan | Ditto | Ditto |
| Rosa Ellen Thaxter | Central Home, London | New Malden |
| Marie Jones | Northampton | Northampton |
| Alice Beaumont | Portsmouth | Portsmouth |
| Amelia Thompson | Cardiff | Quedgeley |
| Edith M. Fechtmann | Bermondsey | St. Helen's |
| Caroline M. Firminger | Battersea | Ditto |
| Catherine West | Hammersmith | Ditto |
| Florence A. Bell | Chelsea | St. Austell |
| Helen Reid | Salford | Salford |
| Sarah E. Robinson | Ditto | Ditto |
| Catherine H. Rudd | Ditto | Ditto |
| Jane A. Jones | Shoreditch and Bethnal Green | Silvertown |
| Annie E. Howard | Camberwell | South Bedford |
| Wilhelmina Irwin | Battersea | Stamford |
| Margaret Modlin | Tunbridge Wells | Tunbridge Wells |
| Margaret Chexfield | Brighton | Whitehaven |
| Alice E. Stevens | Central Home, London | Whitwell |
| Gertrude Mitchell | Windsor | Windsor |
| Dorothy Salt | Ditto | Ditto |
| Christmas Prentice | Brighton | Woolwich |

WALES.

| | | |
|---|---|---|
| Sarah Roberts | Liverpool | Aberystwith |
| Mary Coverdale | Cardiff | Cardiff |
| Alice Mary Darrah | Shoreditch and Bethnal Green | Conway |
| Anna Jones | Salford | Dolgelly |
| Margaret M. Jones | Walworth | Morriston |

SCOTLAND.

| | | |
|---|---|---|
| Margaret J. Farquhar | Edinburgh | Edinburgh |
| Sarah Boyce | Ditto | Ditto |
| Janet G. Christie | Ditto | Ditto |
| Mary C. Day | Ditto | Ditto |
| Elizabeth F. Macleah | Ditto | Ditto |
| Mary P. B. Sandison | Ditto | Ditto |
| Isabella Watson | Ditto | Ditto |
| Barbara Bell | Ditto | Anna |
| Jean McGeorge | Ditto | Blairgowrie |
| Jane Duncan | Ditto | Cambuslang |
| Sarah Longson | Ditto | Dundee |
| Annie R. Borthwick | Ditto | East Wemyss |
| Katherine Carmichael | Ditto | Glendaruel |
| Agnes L. Eldred | Ditto | Larkhall |
| Mary M. Stoddart | Ditto | Ditto |
| Mary Campbell | Ditto | Lochbuie |
| Gertrude Ashby | Ditto | Montrose |
| Isabella Acheson | Ditto | Paisley |
| Margaret Strang | Ditto | Perth |
| Annie Macleod | Ditto | Tobermory |
| Jessie Scott Gray | Ditto | Wishaw |
| Martha Hill | Glasgow | Glasgow |

IRELAND.

| | | |
|---|---|---|
| Jane Simpson | St. Patrick's, Dublin | Ballymena |
| Nannie Hassett | St. Lawrence's, Dublin | Castlebellingham |
| Louisa Hogarth | St. Patrick's, Dublin | Crumlin |
| Alice Mary Hipsey | St. Lawrence's, Dublin | Dublin |
| Mary Ellen O'Reilly | Ditto | Ditto |
| Olive Maria Keane | St. Patrick's, Dublin | Ditto |
| Grace Simpson | Ditto | Ditto |
| Elsie Wallworth | Ditto | Ditto |
| Mary E. O'Keefe | Valencia Island | Valencia Island |

### Where to Go.

ROYAL BOTANICAL GARDENS, REGENT'S PARK, FRIDAY, JULY 4.—1.30 till 11 P.M., garden fête, concert, and pastoral play in aid of the special appeal for St. George's Hospital.

### TRAVEL NOTES AND QUERIES.

STEAMERS FROM THE TOWER BRIDGE (A. M. A.).—The address you want is the General Steam Navigation Company, 33 Great Tower Street, London. They will give you all particulars.

ACCOMMODATION IN SARK (A. M. S. R.).—You give me no pseudonym but I hope you will see this. Look in next issue, and I hope to be able to tell you just what you want to know. I have a friend there who can give me information. I think it would be cheaper and far more enjoyable than England.

### Out-patients in the Transvaal.

BY AN ARMY SISTER.

Unlike most branches of hospital work, "our out-patient department" began in quite an unpremeditated manner. A Kaffir boy was run over not far from the building and was brought in for first aid; he proved to be in too bad a condition for removal, and for a time became an in-patient—returning, when able to go to his kraal, occasionally for dressings. One day it fell to the lot of the sister to dress him, and as things turned out she alone in future attended to his case.

### Not a Savoury Assemblage.

There is no doubt that Kaffirs can talk—how volubly, I once learnt by bitter experience when compelled to spend a night in Pretoria Station, in a carriage adjoining a truck load of natives—and the news spread from mouth to mouth that medical treatment was to be had for natives at the hospital. They came by ones and twos at first, gradually the numbers increased, and from 30 to 50 would attend in a morning, and wait patiently for hours till the sister, free from other calls, was able to see her motley black group of men, women, and children. They were not a picturesque party in spite of many-hued blankets—their favourite, and not infrequently, only garment. Ranged against the hospital fence in every sort of attitude, babies of all ages slung over the women's shoulders in the most marvellous manner which to unaccustomed eyes threatened asphyxia or dislocation; little children clad in beads, and men in tattered and filthy European garments; women with clothes of all colours folded turban-like on their heads, and dressed in sombre-hued prints surmounted by every description of blanket—no, they were not a beautiful nor a savoury assemblage. But oriental patience! there they sat or stood in the fierce sun, tormented with flies (we, here, can understand what the fourth plague meant to the Egyptians), tired, yes, very tired often, for the fame of the hospital travelled far, and patients came over 20 miles to our out-patient department—one poor creature was wheeled that distance in a barrow, as she was unable to walk—wretched specimens of humanity with all manner of complaints and loathsome sores—but so patient and trustful of the white man's skill and kindness; English kindness, for there is no love lost between Kaffir and Boer.

### Lupus Patients.

The cases were as much surgical as medical; leprosy, elephantiasis, and other skin diseases of a contagious description predominated, and those not hopeless from the start responded wonderfully to very simple treatment, and, as we could not help feeling, under very adverse circumstances, for the dirty condition of the native must be a very paradise for germs. We had some of the worst cases of lupus I have ever seen—perfectly revolting wounds which made one defer the evil moment of dressing as long as one could, and yet improvement was remarkable. An old fellow without nose, upper lip, or palate became fairly presentable in six weeks. Sister was most anxious that the surgeon should do a plastic operation and supply deficiencies. Gangrenous sores healed quickly, and burns in a way that were simply astonishing. We had frequently to give four or five days' dressings to the patients who lived at a distance and could not attend daily.

### The Language Difficulty.

The difficulty of making oneself understood was real, and by good fortune sometimes there would be a patient who knew a little English and acted as interpreter all round. Failing this, we were reduced to ocular demonstration more graphic than lovely. If you wished a woman to gargle you had to go through the whole performance yourself, and with many gesticulations towards medicine, glass, mouth, pail, and by vocal example make the patient understand to go and do likewise. Or if you desired a mixture taken three times a day it was necessary to drag your patient to the stoep, point to the sun, with the glass and medicine in your hand, in the east, zenith, and west, to signify t.d.s. Kaffirs have a good sense of humour, and much laughing on both sides might usually be heard in the surgery. We learnt a few words of the language, but, to judge by the fun our use of them occasioned, made many absurd mistakes. It had its dangers too, ludicrous ones, nevertheless. A woman complained of a pain in her "moc," which we interpreted as "stomach." Symptoms seemed to point to castor oil as the line of treatment and it was forthwith administered. We afterwards discovered that the woman was suffering the pangs of hunger! We supplied bread in addition, and trusted that she would be none the worse.

### Kaffir Fondness for Medicine.

Ignorance seems to be the starting point for faith in drugs. We all know of bread pills and hypodermic injections of water for insomina; not to mention the R.A.M.C. dictum "you must order medicine for Tommy because he thinks you're doing nothing for him if you don't," which finds a parallel in many an English out-patient department. Kaffirs share this fondness for medicine applied, internally or externally. A bottle of medicine makes a native happy. We had some cough mixture for the babies which the dispenser labelled "Piccanium." Most Kaffir children have coughs, and many have sore eyes, the latter not surprising in this land of dust and flies. In doubtful cases the doctor was called in and ordered what was necessary, but the majority of cases were such as could be treated by a nurse of experience. One of the sisters was most successful with some skin grafting for a large superficial sore, the grafts took splendidly, upsetting a great many of our theories on the subject. Either the native has some property in his system that can resist forces which unfortunately are, with every care, often too much for English surgery; or else South African air has some peculiarly favourable action on wounds.

### No Eggs, no Treatment.

It must not be thought that natives were treated gratuitously. Kaffirs were expected to bring eggs in exchange for services rendered, and appeared to be quite willing to do so. Fowls, too, were sometimes offered and occasionally brought as presents by grateful patients. Now and again we had to insist on, No eggs, no treatment. This was signified by holding out two empty hands, then pointing to the wounds, and with a vigorous shake of the head saying emphatically, "Icona," which means a negative in any context. When it could safely be done, the patient who persistently forgot eggs was sent away without treatment. Four or six were invariably brought next time. We have taken as many as fifty in a morning. They proved most acceptable for the soldiers when not otherwise obtainable. The sisters looked on a fowl as a special tribute to them, and there would be a laughing discussion as to this or that doctor's prior claim on the delicate morsel, for such it was in a desert of trek-ox lunches and dinners. Sometimes the women would present a bangle or a pair of earings to a sister, and, truth to tell, the latter were not above asking for them occasionally when a very novel kind was worn. A tooth-brush has figured as this ornament more than once! The Basuto bangles and collarbands are handsome, and made of solid brass; the Kaffirs make theirs usually of brass, steel, or copper wire, prettily combined. Beads are quite a feature of attire, little babies having a string or two round the waist; these become of heavier make for older children. The mothers seem fond of their children.

### A Baby for a Buckle.

Once we offered a woman who wanted 1s. for a bangle, and seemed ignorant of the existence of any other coin, that sum for the tiny black baby in her arms; it was indignantly refused; but another woman offered her baby to the sister in exchange for a silver buckle she was wearing. This bargain was politely declined. At times the work seemed hopeless. Patients, after most vigorous and lucid exhortations to return next morning, would not perhaps appear for a week or ten days, and then with everything in a hopeless state of dirt, bandages scarcely recognisable from rags, but, strange to say, the wound underneath progressing well.

### The London Hospital Pension Scheme for Nurses.

The Honourable Sydney Holland, Chairman of the London Hospital, writes:—

In your issue of the 21st you write that "it is not to be expected that the Pension Scheme of the Governors of the London Hospital will create any enthusiasm among the nurses for whose benefit it was supposed (this is not very nicely put, is it?—S. H.) to have been framed," and you go on to comment on the fact that after 18 years' service the pension is only given "during the pleasure of the committee."

I did not expect, none of us did, that this scheme would meet the enthusiastic comment of The Hospital, or even your favourable comment. But I think that in fairness you ought to have stated the whole scheme. Our pension fund is in no way antagonistic to the Royal National Pension Fund. On the contrary, when introducing it to the governors, I took the opportunity of praising that fund. But we have found that we, the house committee, have for years past been paying half the annual premiums for those of our nurses who belonged to that fund, and that this did not in any way bind them to us, but simply gave those who joined the fund a sum of money in addition to their pay, which they could, subject to the rules of the fund, take out the moment they liked. Moreover, this money was not given necessarily to the best nurses or to those who wanted money most, but only to those who could afford to belong to the fund.

Now the object of a pension fund, speaking from an employer's point of view, is to reward long service; and this our scheme does, as all will see if you will quote it. Every member of the staff is to get, in addition to the ordinary increments of salary, an addition of £5 a year after six years, and a second increase of £5 a year after 12 years, and at the expiration of 18 years a pension amounting to full pay. It is true, as you say, that the pension is "during the pleasure of the committee," but those saving words are only put in, as everyone knows, to enable the committee to withhold the pension in the very unlikely event of a nurse grossly misbehaving. The pension, like the pay, is one of the terms of employment, and would be the first call on the hospital funds—that is the law. During good behaviour would perhaps have been less likely to have been misunderstood, if the words you quote have been misunderstood.

Only 77 nurses out of our 470 belonged to the Royal National Pension Fund, so it in no way met the pension difficulty, unless we had compelled all to join, which we did not think right. This did not look like enthusiasm.

I have no evidence as to whether our nurses have received our scheme with enthusiasm, but I have been very warmly thanked for it, very warmly indeed, by nurses who have met me since it was published.

Yours faithfully,
Sydney Holland,
Chairman, London Hospital.

Since receiving the above the following memorandum has come to us:—

PENSIONS.

London Hospital, Whitechapel, June 23rd, 1902.

As the committee desire to encourage suitable nurses to remain in the service of the hospital, they have decided to give every member of the nursing staff an addition of £5 per annum to her salary, after six years from the date of her entrance as probationer. This sum will be given whether the nurse in question is fulfilling the post of a sister, staff nurse, or private nurse. After 12 years' service a second increase of £5 per annum will be given. After the expiration of 18 years' service, at a minimum age of 45, all members of the London Hospital nursing staff will be eligible for pensions.

The pension given will be full pay without any allowances. These pensions will be calculated on the actual average pay received during the five years previous to the sister or nurse relinquishing her active connection with the hospital. Pensions are only to be paid during the pleasure of the committee. Any special case not coming under this heading will be considered by the committee on its merits.

[We do not know why Mr. Sydney Holland did not expect an enthusiastic or even a favourable comment from The Hospital. We have always endeavoured to hold an even hand in all that concerns nursing questions; but while supporting what is good we have never hesitated to criticise anything that appears likely to be detrimental to nurses. Now this question of pension lies at the very root of long-service nursing, for if women are to take to nursing not as a mere temporary employment but as a life's work they must be in some way assured as to their income when such work is no longer possible and its emoluments cease.

Security and certainty are of the very essence of a pension. Hence the paragraph of which Mr. Holland complains. But that paragraph was not aimed at the London Hospital; indeed, we may say that, if we could have felt assured that such a scheme as was set forth by Mr. Holland in his speech at the Quarterly Court would not be taken up by other hospitals, we might have said nothing on the subject. But the London Hospital is so great an institution that it does, to a certain extent, set the fashion in regard to many nursing matters, and it would, in our opinion, be a very serious thing indeed for nurses if little hospitals all over the country were to set up separate pension schemes according to which nurses would receive their pensions only "during the pleasure of the committee."

From our knowledge of the many difficulties of a nurse's life, we are much inclined to question the desirability of her pension ever being paid by her employer. Among workers of all degrees it is universally felt that pensions should not be in the hands of those who hold the power of dismissal. But putting all that on one side, we come back to conditions on which the pension is granted. Mr. Holland says: "The pension, like the pay, is one of the terms of employment, and would be the first call on the hospital funds—that is law." No doubt that would be so but for the clause to which we take exception, and one reason for taking exception to it is that it takes away the very security which Mr. Holland says "is law." The clause is absolutely definite: "Pensions are only to be paid during the pleasure of the committee." That is the condition; it is "one of the terms of employment." The committee is left absolutely unfettered, and the security which, as we have said, is the very essence of a pension does not exist.

This matter is of vast importance to hospital nurses. Let Mr. Holland for a moment forget the London Hospital—a difficult task, no doubt, for one who has so identified himself with all its interests—and let him consider what might and surely would happen at other and smaller hospitals. Committees change, and although at the present time sympathy with nurses and their wants is manifest on every side, it may not and probably will not always be so. In thirty or forty years' time, when the pensions will become a serious drag upon impecunious hospitals, the fact that they are "only to be paid during the pleasure of the committee" will doubtless appeal with considerable force to some enterprising committeeman. What Mr. Holland says about the system of the hospital paying half the annual premium to the National Pension Fund is also, we think, a matter of much importance and worthy of careful consideration.

It is clear that the money so paid does "not in any way bind" the nurses to the hospital, which "from an employer's point of view" is one object of a pension fund. But then we do not look at the matter entirely from the employer's point of view. From the nurse's point of view we have no hesitation in saying that there must be greater liberty, less subserviency to those in authority, and far greater security in a National Fund than in a local arrangement.—Ed. The Hospital.]

### Everybody's Opinion.

[Correspondence on all subjects is invited, but we cannot in any way be responsible for the opinions expressed by our correspondents. No communication can be entertained if the name and address of the correspondent are not given as a guarantee of good faith, but not necessarily for publication. All correspondents should write on one side of the paper only.]

### DERBYSHIRE NURSING INSTITUTE.

"EDWARD S. JOHNSON," the secretary of the Derbyshire Nursing Institute, writes: Thank you for your kind notice in this week's issue of THE HOSPITAL, *Nursing Section*, of our annual report. As the figures given are somewhat misleading, may I be allowed to explain that the deficiency to which you refer on our general account was £2 16s. 8d., against which we had the interest from our investments. You will see by the enclosed that our balance to credit on the year was £157 19s. 3d.; this we distributed as a special bonus, in which our district nurses participated. We have anticipated your suggestion, having decided to place another nurse on our district staff.

### AN APPEAL FROM A PRIVATE NURSE.

"NURSE MARGUERITE" writes: Cannot something be done to give the private nurse a little pleasure, or to make her life somewhat brighter? I have charge of a chronic; am on duty from 9 A.M. till 9 P.M. (when I am relieved by the night nurse), with no time off duty, my only time, therefore, for exercise being at night, when I do not care to wander about alone, and I have no friends near. Having done several months of nursing, I am feeling the strain too much, and yet I must try to keep up! Will some philanthropically inclined person try to start an agitation for the benefit of poor private nurses whose employers exact their full pound of flesh!

### NURSES AND RELIGION.

"ADA" writes: May I be allowed to say in reference to the last clause in "R. H. E.'s" remarks, that I hope the time will never come when the question of the would-be nurse's religion is omitted. Nursing and religion, as we all know, have from the earliest times always been associated. Every nurse must know what denomination she was baptized in and can consider herself a member of. But as the Church of England is our National Church, I do not think that any nurse in our hospitals should object to attend its services when they are held in the building in which she is employed.

"NURSE MAUDE" writes: May I be allowed to make a few remarks on the subject of "Differences in Religion"? The difficulty a Roman Catholic nurse has in procuring a post at a hospital or private home is surprising to me. I have known several cases of nurses with excellent qualifications being rejected with a curt note, "Must be Protestant." Is religion part of the training? Surely this is a matter of the nurse's own conscience! Catholics and Protestants all worship the same God, all having the same aim in view. What are the objections? Should a patient be dying under the care of a nurse of either Church, she should be prepared to talk to her, according to the creed of the sick person, without feeling bigoted. Therefore if a nurse does not interfere with the religion of those about her, attends to the comfort of the sick, in all thoroughly does her duty, why should this talented worker be dismissed? Many Catholics help to support the hospitals and do a great deal toward the relief of the suffering in the nursing world. I do not believe it is always the fault of the matron that the objection is made, but in some cases hospital authorities have framed the rule out of real ignorance. Perhaps it would be wise to encourage an unselfish and Christian feeling for the welfare of many nurses who are often out of work on account of this system.

"P. E. T." writes: May I be allowed, in answer to "R. H. E.", to point out that, "having no creed," she could surely raise no objection to attending morning and evening service in the infirmary church on Sunday, as from her letter it is evident that no interference is made regarding the nurses' religious views? I think "R. H. E." will see the impossibility of any matron allowing several nurses to attend individual places of worship, for their times of duty and the distances would cause much friction. I know that Roman Catholic nurses often refuse appointments where they are unable to get church privileges, but it seems incredible that any nurse should consider it too much to devote a little time one day a week to prayer. What would "R. H. E." do in an institution in which morning and evening prayers are the rule? I am only too pleased to say that in the union infirmary where I am we never miss saying them, and most of the patients join in. Surely a nurse with no religion could hardly be expected to do her work from the highest motives. As for a "conventional religion" there is no such thing as far as I know. It is true that there are many nurses and others who go to church because it is the rule, or the fashion, and who care not at all about it, but surely it is themselves who are to blame and not the religion. I think if "R. H. E." could not give a clergyman's reference, but stated that she was quite willing to attend the same place of worship as the other nurses, she would find no difficulty in being accepted as a probationer. I remember that while I was at a Roman Catholic school abroad I had to attend the services with the rest of the pupils. I think there are certain times when we should "do as Rome does" or why the proverb?

### ENGLISH NURSING IN ROME.

"M. F. D." writes:—My attention has just been called to an article in your issue of June 14th entitled "English Nursing in Rome." Had your correspondent been content to write about her own home I should have said nothing. But her most unjust and unwarrantable insinuations against the English nuns have disgusted everyone. To begin with, it is absolutely false to say that the sisters do not speak the language, many being highly accomplished ladies, speaking French, German, and Italian with ease. Can your correspondent do the same? All the sisters who undertake cases are certificated, and in infectious cases the habit is exchanged for a cotton one with linen veil. Again, it is most inaccurate, to use the mildest word, to say that there is only one community of nuns who nurse. There are several of various nationalities; but the one which your correspondent evidently aims at—the English one—has been established over twenty years in Rome, and seen the downfall of two Protestant and secular nursing institutes. Only recently a Protestant lady sent away a secular nurse for requiring 15 out of the 24 hours to herself! It is too absurd to talk of one nurse at this place or that. There were within the last four weeks six sisters summoned from Rome and Florence to the most fashionable watering place in Italy, where the head doctor is a Protestant. Your correspondent mentions with pride a staff of "eight nurses." The sisters have twenty constantly employed, to say nothing of the French and Swiss sisterhoods whose houses are filled to overflowing. To me, an English resident (and a secular for the last twenty years), it would be amusing were in not so unjust to the noble sisters who devote their lives to the sick of all creeds and nationalities. In common fairness to Catholics I trust you will insert this, for I may add it is not the first time you have been obviously unjust to Catholics. In conclusion, I should say that too many "trained nurses" spend their small savings on a journey to Rome, only to be assisted home by charity.

### A HOME FOR PRIVATE NURSES WANTED.

"B." writes: I feel rather indignant at the complaint of "A Private Nurse," that at the Nurses' Hostel, in Francis Street, you cannot get food except at meal hours, and also as to the want of comforts. Why should nurses, any more than any other profession, expect to get the best living and comforts at the price of the cheapest? Nurses, as well as other workers, know within a little the probable income which they can earn, and must look ahead and suit their living to their purses; of course we do not like it, but workers have to be content to "do without" a great deal that many regard as necessaries. I have been a nurse for many years, and have often made use of the hostel, and am always struck with the amount one gets for one's money there. I am quite sure that one could not live for less anywhere, especially with all the conveniences of electric light, hot and cold baths, a quiet room for writing, a nice sitting-room, with sofas, easy chairs, books, and the daily papers. Some nurses are so very ready to demand luxuries as their right, but how many are there who are ready to pay for them? No institution can ever meet the wants and wishes of all, because when everyone pays, everyone says, "Me first." Think of any family home, where there are several sisters, and what happens when each wants the daily paper or the sofa, or a particular chair or corner, and their tastes in regard to food, light, air, are so varied. Must there not be giving and taking, bearing and forbearing? But in an institution the very fact of payment gives each a sense of injury if her own wants, wishes, or conveniences are not met. I am afraid that by a comfortable home "A Private Nurse" means a place where one can do exactly as one wishes; have meals how, when, and where one pleases, and in fact every comfort without any responsibility. Has "A Private Nurse" ever had the management of any household? does she know what meals at odd hours entail on the housekeeper and maids? Surely the small cost of living at the hostel will admit of a nurse keeping a spirit lamp and kettle, and providing herself with the necessaries for a breakfast or supper—I know that milk can be had from the dairy opposite—and you do not pay the hostel for meals which they do not provide. Let "A Private Nurse" count up all the expenses of house-rent, etc., as well as service and food. I think she will find that at the hostel in Francis Street you get more than full value for your money. I have not seen the new buildings, so I am only speaking from my experience of the other block.

### Presentations.

NATIONAL HOSPITAL FOR CONSUMPTION FOR IRELAND, NEWCASTLE, WICKLOW.—The nursing staff and hospital employés of the National Hospital for Consumption for Ireland, have presented the resident medical officer, Dr. B. H. Steede, with silver-plated dinner and dessert forks on the occasion of his marriage to Miss Maude Powell, of Dublin.

WEST LONDON HOSPITAL, HAMMERSMITH. — Miss L. Gaved-Wills, who has just been appointed lady superintendent of the Newark Hospital and Infirmary, has been the recipient of many valuable gifts from the matron and nursing staff of the West London Hospital, of which she has been a sister for the past six years.

### Wants and Workers.

THE matron of Dockwell Hospital, near Hounslow, would be grateful for old linen for dressings and handkerchiefs for smallpox patients.

### To Nurses.

**WE invite contributions from any of our readers, and shall be glad to pay for "Notes on News from the Nursing World," or for articles describing nursing experiences, or dealing with any nursing question from an original point of view. The minimum payment for contributions is 5s., but we welcome interesting contributions of a column, or a page, in length. It may be added that notices of appointments, entertainments, presentations, and deaths are not paid for, but that we are always glad to receive them. All rejected manuscripts are returned in due course, and all payments for manuscripts used are made as early as possible after the beginning of each quarter.**

### Appointments.

[No charge is made for announcements under this head, and we are always glad to receive, and publish, appointments. But it is essential that in all cases the school of training should be given.]

BECKETT HOSPITAL, BARNSLEY.—Miss E. R. Roberts has been appointed night sister. She was trained at the Royal Southern Hospital, Liverpool, and St. Paul's Eye and Ear Hospital, Liverpool. She has since been staff nurse in the Royal Southern Hospital, Liverpool, and sister, for eighteen months, of women and children's wards in the Beckett Hospital, Barnsley.

BRADFORD ROYAL INFIRMARY.—Miss A. M. de Ville has been appointed home sister. She was trained at the Victoria Hospital, Burnley, where she was afterwards sister for two years. She has since been for two years sister at the Royal Portsmouth Hospital, night superintendent for eighteen months at the Royal Halifax Infirmary, and night superintendent for eighteen months at the Royal Infirmary, Preston.

BROOKE DISPENSARY AND COTTAGE HOSPITAL, SELBY.—Miss Lily Edginton has been appointed matron. She was trained at Fir Vale Infirmary, Sheffield, where she was afterwards charge nurse. She has since been on the private staff of the Sheffield Nurses' Training Institution. Miss Edginton holds the L.O.S. certificate.

CHIPPENHAM WORKHOUSE INFIRMARY.—Miss Emily M. A. Jones has been appointed superintendent nurse. She was trained at the Royal Infirmary, Edinburgh, and in midwifery at Kensington Infirmary, where she obtained her L.O.S. certificate. She has been doing private and district work for the last six years in connection with the Burton-on-Trent Nursing Institution.

COTTAGE HOSPITAL, LEEK.—Miss R. Mark has been appointed staff nurse. She was trained at Lambeth Infirmary.

EVESHAM WORKHOUSE INFIRMARY.—Miss Edith Sarah Malvern has been appointed superintendent nurse. She was trained at Kensington Infirmary and the City of London Lying-in Hospital, and has since been superintendent at Andover Union Infirmary.

ISOLATION HOSPITAL, SHANGHAI.—Miss Alice Bradford has been appointed matron. She was trained at the Royal County Hospital, Winchester, where she was afterwards sister; she has since been sister and night superintendent at the Royal Infirmary, Southampton, and matron of the Accident Hospital, Mansfield. For the past three years she has been doing private nursing in Mansfield.

NORDRACH-ON-DEE SANATORIUM.—Miss H. M. Murray has been appointed lady superintendent. She was trained as the Royal Infirmary, Edinburgh, and has since been sister at Victoria Infirmary, Glasgow.

NOTTINGHAM CHILDREN'S HOSPITAL.—Miss Helen Simmons has been appointed sister. She was trained at the Children's Hospital, Nottingham, and St. Bartholomew's Hospital, London. She was also on the private staff for a year at St. Bartholomew's, and acted for a short period as assistant housekeeper.

SEAMEN'S HOSPITAL, GREENWICH, S.E.—Miss Isabel E. Reynolds has been appointed night sister. She was trained at the General Hospital, Wolverhampton, and has since been charge nurse at the Grove, Homerton, sister of men's wards and theatre at the Chesterfield Hospital for Accidents, and Chichester Infirmary, sister of men's surgical ward at the General Hospital, Birmingham, and of the men's medical ward at Leicester Infirmary.

### For Reading to the Sick.

### THE LISTENING ANGELS.

Blue against the bluer Heavens
Stood the mountain, calm and still,
Two white Angels, bending earthward,
Leant upon the hill.

Listening leant those silent Angels,
And I also longed to hear
What sweet strain of earthly music
Thus could charm their ear.

When the sunset came in glory,
And the toil of day was o'er,
Still the Angels leant in silence,
Listening as before.

Then, as daylight slowly vanished,
And the evening mists grew dim,
Solemnly from distant voices
Rose a vesper hymn.

When the chant was done, and lingering
Died upon the evening air,
From the hill the radiant Angels
Still were listening there.

Silent came the gathering darkness,
Bringing with it sleep and rest;
Save a little bird was singing
Near her leafy nest.

*A. A. Proctor.*

All great things come in silence. For often silence implies power without parade. It is an outcome of those vast forces of passive virtue which are the strongest things in the soul.

In silence we come by our truest thoughts; in silence we form our wisest and strongest resolves; in silence we feel our bitterest pangs of sorrow; in silence we drink our deepest draughts of joy; in silence we touch the depths of repentance; in silence we feel the real blessedness of peace. Yes, and sympathetic souls often in silence hold intercourse in that unspoken language, which is the channel of truest interchange of thought from soul to soul.

Christ came in silence. To the world at large, amid the hubbub of the cities, and the cries of sufferers, and the laughter of the boisterous, and the constant noise and movement of an ordinary working world, no ear could catch the tread of the unseen feet, and hear Him come.

Only He *had* come. There He was, the second Adam, the Chief and Reviver of a ruined race. To the poor and simple, the few watchers and waiters, He was known; but deep had been the silence of the coming, and the world could not realise His awful Presence. "He had come down like rain into a fleece of wool, even as the drops that water the earth."

*Knox-Little.*

So when the tones of rapture gay
On the lone ear die quite away,
The lonely world seems lifted nearer Heaven;
Seen daily, yet unmarked before
Earth's common paths are strewn all o'er
With flowers of pensive hope, the wreath of man forgiven.

*Keble.*

### The Nurses' Bookshelf.

Lights and Shadows in a Hospital. By Alice Terton. (London: Methuen and Co. Price 3s. 6d.)

Our first thought on reading this book is, "In what antediluvian days can its author have been trained?" She writes of wearing a linsey gown in her first hospital; of calling her matron "Ma'am;" of taking a casual night on night duty, quite at haphazard, though, from her own showing she was a raw probationer; and she addresses the sister of her ward in a way which would make a modern nurse shiver! And yet she talks of having been trained in the "good old days, when training was really a time of probation, and their (the probationers') humble position was impressed upon them!" Has she really never been inside a hospital of to-day? And does she really not know what a very humble position the modern probationer occupies, nor how great a gulf yawns between that probationer and the sisters?—a gulf, which in the writer's days, would seem to have been bridged over with astonishing ease. We confess to a slight feeling of irritation at her assumption that the large London hospital does not encourage its nurses to make the patients their first consideration, whilst at Highchester (the county hospital with the thinly-disguised name where Mrs. Terton received her chief training), "it was a great joy to find we were always encouraged to be kind to the patients." This is misleading to the lay reader, to whom the book will mainly appeal, for the "lights and shadows" of hospital life are shown chiefly in anecdotes of patients, some amusing, some pathetic, all of more interest to the non-professional than to the professional reader. Part of the book deals with the management of a cottage hospital, and this portion is distinctly good. Many valuable hints might be gleaned from it by cottage hospital matrons. The writer's arrangements were excellent, and her "family," as she calls her household, must have been a happy one. Some of the stories told are excellent, but we have only space to quote one. A publican who was said to be dying, rallied slightly, and his wife was told of the joyful change in his condition. "I call it too bad of the doctors," was her comment, "to mislead one like this. First, he wasn't to get better, and now he is, and I have brought a clean shirt all ready to lay him out, and I've sold the business, and whatever am I to do now? I think it's shameful of the doctors showing one no consideration."

Sick-Room Cookery and Delicacies for Convalescents: a Book of Suggestions. "The Home Series," edited by Florence White. No. 9. (London: Florence White, Howard House, Arundel Street, W.C. Price 2d.)

This little book contains nothing specially new, but it gives a large number of recipes suitable not only for invalids but also for those in good health who have a liking for dainty dishes. The directions given for the preparation of these are clear and practical, and at the moderate price at which it is published there is no reason why it should not be widely popular. It is worth far more than it costs.

A Manual for Assistants' Examination, Apothecaries' Hall. By Mabel F. Stanley. (London: Henry Renshaw. 1902. 281 pages. Price 3s. 6d.)

The manual before us has been written with the purpose of assisting students who are studying for the Assistants' Examination of the Society of Apothecaries. Its scope is limited precisely by the requirements of the syllabus drawn up for that examination, and the subjects have been arranged strictly in the order required. This little volume is a model of careful compilation and well calculated to fulfil the purpose which it is intended to subserve.

### Echoes from the Outside World.

### The King's Illness.

When first it was rumoured on Tuesday last week that His Majesty was undergoing an operation, that his condition was serious, and the Coronation was to be postponed, the statement was generally disbelieved, principally because the King had been seen in public the day before, and most of those who had witnessed his arrival in London had not thought that he looked otherwise than well. But when the official bulletin was published there no longer remained room for doubt, and it is no exaggeration to say that the whole nation was, for the time, paralysed. The fact that within two days of the crowning of Edward VII., when celebrities from all parts of the world were assembled in the capital, the central figure of the ceremony—which was to surpass in splendour all that had gone before it in any century—had been stricken with dangerous illness, seemed impossible to realise. The streets which the procession would have traversed were as full of sightseers as ever, but they were strangely quiet as they passed along, the nearly finished stands were devoid of workmen except in a few instances, and only one subject of conversation was heard on every side. During the night there were several watchers outside Buckingham Palace, and at the time when it was known fresh reports would be published a large crowd always assembled. The same silent expressions of the sympathy and anxiety of the nation were apparent each day and night, not only in London, but in all parts of the world, for the worst was feared till Friday, when hope began to find a firmer footing in the minds of the people. Intercessions had been offered in all churches and chapels throughout the kingdom on Thursday in place of the Coronation service, and were repeated on Sunday, on which day the King was announced to be out of immediate danger. A very welcome evidence of the progress of the illustrious patient was afforded on that morning by the fact that the Queen and all the members of the Royal Family attended service at 10.30 at the Chapel Royal, St. James's. The National Anthem was sung kneeling at the end of the service.

### The Queen at the Reviews.

The Review of Colonial troops held by the Prince of Wales on Tuesday on the Horse Guards Parade was most successful, and the deafening cheers which greeted the progress of the carriage containing the Queen must even have penetrated to the sick chamber in Buckingham Palace. Directly after the Royal Salute Her Majesty gave orders that her carriage should be driven along the lines, and she was accompanied by all the foreign representatives. The Duke of Connaught, the Duke of Cambridge, and Lord Roberts were all present, and the South African contingent were given a specially hearty welcome by the onlookers of the march past. The review on Wednesday morning of the Indian soldiers, by the Prince of Wales, was another great spectacle, and excited a remarkable amount of interest. The reception accorded to the Queen was quite as enthusiastic as on Tuesday, hats, handkerchiefs, and sticks being waved, while the cheering all along the Mall was tremendous. The spectacle presented by the Bengal Lancers, Bombay Grenadiers, Madras Pioneers, Sikhs, Goorkhas and other troopers was gorgeous in the extreme.

### Orphan Children at Marlborough House.

Although for most of the King's subjects, June 26th and 27th lost all the glory which in anticipation had hung around those dates, 2,500 of the orphan children of London will always look back to that Thursday and Friday as days of supreme happiness. Owing to the kindly thought of the Prince and Princess of Wales, the children who had been invited to Marlborough House were bidden to come just the same, although there would be no procession to view. The head master of the Police Orphanage at Twickenham stated that it was one of the most painful tasks he had ever fulfilled when he had to tell his little charges of the King's serious illness and the postponement of his crowning. "After evening prayers" he said, "I called upon them to sing 'God Save the King,' and in their disappointment they were unable to finish it for crying. On Wednesday, however, when I told them at dinner time that they were after all to go to Marlborough House, the heartiness with which they sang would have done anyone good to hear." The children came in two detachments, those from the London Orphan Asylum, the Merchant Seamen's Orphanage, the Police Orphanage, the Royal Asylum for the Deaf and Dumb Poor, the Soldiers' Daughters' Home, and the Royal Caledonian Asylum, arriving the first day; and the little ones from the Foundling Hospital, the Sailors' Orphan Girls' School, Princess May Village Home, Patriotic Fund Orphanage, Soldiers' Daughters' Home, Post Office Orphanage, and Newport Market Refuge partaking of the Royal hospitality on the second. The Prince and Princess of Wales with Prince Edward and Princess Victoria were present both days among their little guests, and seemed extremely pleased that they appeared so thoroughly happy.

### Coronation Honours.

By special command of the King the appointments which had already received his Majesty's approval were made public on Thursday. They include seven new peers, namely the Lord Justice General of Scotland, Sir U. K. Shuttleworth, M.P., Mr. W. L. Jackson, M.P., Mr. Arthur Smith-Barry, General Sir Francis Grenfell, Sir Francis Knollys, and Mr. A. B. Freeman-Mitford. The Earl of Hopetoun becomes a marquis, and Lord Colville of Culross, Lord Churchill, and Lord Milner, viscounts. Among the new K.C.B.'s are Mr. Leslie Stephen, the Hon. Schomberg McDonnell, private secretary to the Premier; Mr. J. L. Gorst, Financial Adviser to the Egyptian Government; and Professor William Ramsay, F.R.S. Among the new baronets are the Lord Mayor of London, Sir Edward Bradford, Chief Commissioner of Police; Sir Edwin Poynter, P.R.A., and Sir Hubert Parry. The long list of knights includes Mr. F. C. Burnand, editor of *Punch*; Dr. A. Conan Doyle, Mr. John McDougall, Chairman of the London County Council; Mr. Gilbert Parker, M.P., the well-known novelist; Mr. W. J. Soulsby, C.B., private secretary to the Lord Mayors of London for more than 20 years; Mr. C. Villiers Stanford, Mr. John Thorneycroft, F.R.S., Mr. Ernest Waterlow, A.R.A., President of the Royal Institute of Painters in Water Colours; Mr. J. L. Wilkinson, general manager of the Great Western Railway; and Mr. Charles Wyndham, the popular actor.

### The New Order of Merit.

The most notable feature of the Coronation Honours was the announcement of the creation of a new Order of Merit by the King, and the appointment to it of twelve of the most eminent persons in the country. The Order, though making provision for taking into its ranks distinguished foreign personages as honorary members, will be principally for British subjects who have won conspicuous distinction in the naval and military services, and for those who are exceptionally eminent as men of letters and in the fields of art and science. The badge of the Order will consist of a cross of red and blue enamel of eight points, having the words "For Merit"—the motto of the Order—in gold letters within a laurel wreath on a blue enamel centre. The reverse of the badge will show the King's Royal and Imperial cipher in gold also within a laurel leaf, on a blue enamel centre; and the whole will be surmounted by the Imperial Crown enamelled in colour, and suspended by a parti-coloured ribbon of Garter blue and crimson, two inches broad. The first recipients of the new Order are Lord Roberts, Lord Wolseley, Lord Kitchener, Lord Rayleigh, Lord Kelvin, Lord Lister, Admiral Sir Henry Keppel, Mr. John Morley, M.P., Mr. W. E. H. Lecky, M.P., Admiral Sir E. H. Seymour, Sir William Huggins, and Mr. G. F. Watts, R.A.

### Notes and Queries.

**The Editor is always willing to answer in this column, without any fee, all reasonable questions, as soon as possible.**

**But the following rules must be carefully observed:—**

1. **Every communication must be accompanied by the name and address of the writer.**
2. **The question must always bear upon nursing, directly or indirectly.**

**If an answer is required by letter a fee of half-a-crown must be enclosed with the note containing the inquiry, and we cannot undertake to forward letters addressed to correspondents making inquiries. It is therefore requested that our readers will not enclose either a stamp or a stamped envelope.**

*America.*

(107) 1. Can you tell me if there is any infectious hospital in New York similar to the M.A.B.'s in England? 2. Also if nurses are scarce in America, and if there is a paper similar to The Hospital published there?—*A. B. C.*

There is no board constituted as our Metropolitan Asylums Board in New York. 2. Nurses are plentiful and well organised in America, and they are represented by a paper called the *Trained Nurse*, which may be obtained at the office of the Scientific Press, Southampton Street, Strand, W.C. Training is very much on the lines of the best English models.

*Endowed Bed.*

(108) Will you kindly tell me if it is the exception for a Cottage Hospital to have an endowed bed?—*N. A.*

Cottage Hospitals frequently have endowed beds.

*Water-bed.*

(109) Will you kindly inform me as to the best way to fill a water-bed? A hospital nurse tells me that I should fill it with the patient on the bed.—*F. M. L.*

The art of filling a water-bed lies in adjusting the quantity of water to a nicety. It must be full enough to support the patient comfortably, yet not so full as to be hard. Fill it about three parts full at first and then add or draw off the water until it is perfectly comfortable.

It is very difficult to move a large water-bed when full of water without tearing it. Hence the proper way is to place the water-bed where it is to lie and then to fill it. If the patient is helpless and unable to be moved out of bed, it may be necessary to fill it while the patient lies upon it, and in many cases it is desirable to do so because it is next to impossible to ascertain the precise quantity of water required except by actual trial. By holding up the corner of the bed to which the pipe is attached it is always possible with a fair sized bed to obtain sufficient "head" of water to float the patient up. To ascertain the exact quantity of water required one must gently insinuate the hand under the water-bed, the hand lying quite flat, with the palm upwards and the finger tips just under the sacrum of the patient, or if the patient has to be propped in an elevated position, under the point of greatest pressure. So long as the finger tips so placed can feel that the sacrum, or other part lying above them, presses the two sides of the bed together so that no water intervenes, more water is required, but as soon as the finger can feel that a layer of water, perhaps a quarter or half an inch thick, intervenes, so that the finger tips have to be slightly lifted to feel the patient's body, then it is clear that the patient is floating, that all her bony points are raised clear of the under surface of the bed, and that there is enough water. The addition of any more water does no good, and only makes the bed feel hard. A small water-bed or pillow must be filled before the patient is placed upon it, unless it is provided with a tube long enough to supply a proper head of water, which is not often the case. It is to be noted that the presence of much air in a large water-bed is an evil, as it causes unpleasant splashing sounds and gives the patient an uncomfortable sense of insecurity. But in a small water-bed a certain amount of air is an advantage from the sense of elasticity which it gives.

*Salsomaggiore.*

(110) Will you kindly tell me if there are any English doctors at Salsomaggiore, and if so, give me their addresses. Is their a nursing institute?—*Dun Spiro Spero.*

Consult a medical directory. There is no nursing institute that we know of. There is generally an English chaplain at foreign health resorts during the season; a courteous note, enclosing stamps for reply, would very likely bring you reliable information on the subject.

*Concentration Camps.*

(111) To whom should I apply for information as to nurses for the Concentration Camps in South Africa? I have had three years and some months' training, and am desirous of offering my services.—*Nena and M. R. D.*

The war being ended, it is scarcely likely that more nurses will be required.

*Child s Nurses.*

(112) Will you be good enough to tell me if you know anything of the Norland for Nurses, and can you give me the address?—*C. I. W.* (Southport).

The Norland Institute is designed to train ladies for the duties of child's nurses. Apply to the Secretary, 10 Pembridge Square, London, W.

Will you kindly let me know if there is such an institution as the Norland Institution where ladies are trained as children's nurses?—*Nurse W.*

See reply to *C. I. W.* (Southport).

*Maternity.*

(113) I should be grateful if you will advise me what to do under the following circumstances. I was engaged to attend a case on June 1st. I was called to it a month beforehand, and gave up a night case which I was attending in order to go. On the fifth day my patient developed puerperal septicæmia through no fault of mine. The doctor then told me that I must give up a case I had for July as I must have a month's quarantine, and I and my clothing, instruments, etc., be thoroughly disinfected. I accordingly gave up my July case, and on the seventeenth day I broke down in consequence of losing my rest day and night, my throat becoming bad from breathing the impure air. I had help for three nights in the middle of the case, but the work was so objectionable that the other nurse would not stay, and I had again to take all the duty myself. The question is what fees can I claim? Can I claim the month's money; and am I entitled to any compensation for my month's quarantine? Or can I only ask payment for the seventeen days I nursed the case? It seems very hard that I should lose in every way, but I do not wish to be extortionate.—*Semper Fideles.*

It is a very unfortunate case as you cannot claim anything except for the seventeen days of actual work. However, as through no fault of yours, or indeed of anyone, you have been so heavy a loser most probably your employer would be willing to make you some reasonable compensation, say half fees for your month of quarantine, if the matter were rightly represented.

Will you kindly tell me what are the usual fees charged per week in London for a thoroughly experienced monthly nurse, and also say if it is usual to reduce the fee should her services be required for a longer period either before or after the month?—*S. S.*

The usual fees for a fully-trained nurse—that is a nurse with certificate for general training as well as maternity—are £2 a week, £8 for the four weeks. It is entirely a matter for arrangement as to whether the fees are reduced or not.

*Uniform.*

(114) Will you kindly tell me if a certificated mental nurse may wear uniform?—*M. H.*

Certainly.

*Replies to Correspondents.*

(115) No notice whatever has been taken of my letter addressed to the secretary, in which was enclosed a stamped envelope for a reply. I therefore repeat the request therein contained, and would feel obliged for an answer without further delay. "Please forward the address of a nursing home from which could be obtained capable hospital-trained women for non-infectious cases for the sum of one guinea weekly."—*A. de B.*

Will A. de B. kindly read our rules to correspondents at the head of this column? No capable hospital-trained woman can be obtained for one guinea per week for the simple reason that they can get twice that sum.

*Holidays.*

(116) Will you kindly tell me what holidays I am entitled to under the following circumstances? I began duty many years ago, and, having resigned, my engagement terminates on May 3rd. My agreement expressly stated that my holidays were to be not less than one month in each year, and I have not had a single day.—*Nurse Rhoda.*

You made a mistake in not claiming your holidays as they became due. It will be exceedingly difficult to enforce any claim now that you have resigned, and your best plan seems to be to represent the matter courteously to your committee and ask for a grant in consideration of your long services without holidays.

**Standard Books of Reference.**

**"The Nursing Profession: How and Where to Train." 2s. net; post free 2s. 4d.**
**"Burdett's Official Nursing Directory." 3s. net; post free, 3s. 4d.**
**"Burdett's Hospitals and Charities." 5s.**
**"The Nurses' Dictionary of Medical Terms." 2s.**
**"Burdett's Series of Nursing Text-Books." 1s. each.**
**"A Handbook for Nurses." (Illustrated). 5s.**
**"Nursing: Its Theory and Practice." New Edition. 3s. 6d.**
**"Helps in Sickness and to Health." Fifteenth Thousand. 5s.**
**"The Physiological Feeding of Infants." 1s.**
**"The Physiological Nursery Chart." 1s.; post free, 1s. 3d.**
**"Hospital Expenditure: The Commissariat." 2s. 6d.**

**All these are published by the Scientific Press, Ltd., and may be obtained through any bookseller or direct from the publishers, 28 and 29 Southampton Street, London, W.C.**

